# The Role of TRAF6 in Cancer: From Molecular Mechanisms to Therapeutic Strategies

**DOI:** 10.3390/cells15090818

**Published:** 2026-04-30

**Authors:** Shuai Xiao, Dandan Song, Yeping Yu, Lingli Tian, Xiaozhen Xu, Wenying Qin, Rui Zhang, Hao Lyu, Dong Guo, Qi Zhang, Xing-Zhen Chen, Jingfeng Tang, Cefan Zhou

**Affiliations:** 1 National “111” Center for Cellular Regulation and Molecular Pharmaceutics, Hubei University of Technology, Wuhan 430068, China; 2 Henan Key Laboratory of Tea Plant Biology, College of Tea and Food Science, Xinyang Normal University, Xinyang 464000, China; 3 Protein Disease Research Group, Department of Physiology, Faculty of Medicine and Dentistry, University of Alberta, Edmonton, AB T6G 2R3, Canada

**Keywords:** TRAF6, ubiquitination, tumor, NF-κB signaling, therapeutic target

## Abstract

**Highlights:**

**What are the main findings?**

- The review summarizes the biological functions and oncogenic roles of TRAF6, highlighting its E3 ubiquitin ligase activity in catalyzing K63-linked polyubiquitination of key substrates and its aberrant overexpression across various malignancies.
- It clarifies the molecular mechanisms by which TRAF6 facilitates cancer progression, involving the activation of NF-κB, MAPK and PI3K/AKT cascades, inhibition of apoptosis, and a systematic overview of current TRAF6-targeted therapeutics.

**What are the implications of the main findings?**

- The close correlation between abnormal TRAF6 expression and poor clinical prognosis suggests its potential as a prognostic biomarker for malignancies, aiding in patient stratification and treatment decision-making.
- Targeting TRAF6 offers novel and promising therapeutic avenues. Tumor-specific delivery and combination strategies are expected to optimize anti-tumor efficacy and alleviate non-specific side effects.

**Abstract:**

Tumor necrosis factor receptor-associated factor 6 (TRAF6) is an E3 ubiquitin ligase that plays a crucial role in inflammation, immune responses, and tumor development. It was reported that TRAF6 primarily catalyzes K63-linked polyubiquitination to stabilize substrate proteins, thereby facilitating the malignant phenotype of tumors. Beyond its cytoplasmic roles, TRAF6 undergoes nuclear translocation in response to specific stimuli, where it interacts with chromatin modifiers. TRAF6 acts as a central mediator in key signaling pathways downstream of the Toll-like receptor, interleukin-1 receptor, and tumor necrosis factor receptor superfamilies, including NF-κB activation. TRAF6 exerts diverse oncogenic functions, including promoting cell proliferation, migration, metastasis, immune evasion, and therapy resistance. This involves modulating cellular pathways such as NF-κB and MAPK signaling, which contribute to malignant progression. Aberrant TRAF6 activation contributes to the pathogenesis of multiple malignancies, including colorectal cancer, melanoma, hepatocellular carcinoma, and acute myeloid leukemia, making it a promising therapeutic target for cancer treatment. This review summarizes the structural features, substrate diversity, and multifaceted roles of TRAF6 in cancer, as well as the development of TRAF6-targeting drugs and strategies. We hope this review can provide a comprehensive perspective on TRAF6-targeted therapeutic strategies for cancer.

## 1. Introduction

Tumor necrosis factor receptor-associated factor 6 (TRAF6), a member of the TRAF protein family, functions as an E3 ubiquitin ligase that plays pivotal roles in multiple signal transduction pathways [1]. It was initially identified as a signaling mediator downstream of tumor necrosis factor receptor 2 (TNFR2) and CD40 receptor [2]. Distinct from other TRAF family members, TRAF6 possesses a unique ability to catalyze K63-linked polyubiquitination, which is critically involved in the activation of key oncogenic signaling pathways [3]. Subsequently, TRAF6 has been recognized as a central adaptor molecule in both Toll-like receptor (TLR) and interleukin-1 receptor (IL-1R) signaling pathways, where it orchestrates essential innate immune responses [4,5]. Notably, recent evidence demonstrates that following IL-1β stimulation, TRAF6 undergoes nuclear translocation and participates in chromatin remodeling through interactions with corepressor complexes [6]. Its dysregulation correlates with tumor progression, metastasis, therapy resistance, and immune evasion across carcinomas, glioblastoma (GBM), and hematologic malignancies [7,8,9]. Given its pleiotropic roles in oncogenic signaling, immune regulation, and subcellular dynamics, TRAF6 represents a mechanistically compelling and therapeutically actionable target for precision oncology.

Accumulating evidence indicates that TRAF6 is overexpressed in various tumor types and plays critical roles in tumor development and progression [10]. Mechanistic investigations reveal that the context-dependent oncogenic functions of TRAF6 are mediated through distinct molecular pathways. For instance, TRAF6 inhibits necroptotic cell death by suppressing the RIPK1/RIPK3/MLKL signaling cascade, thereby promoting the survival of tumor cells [11]. In melanoma, TRAF6 regulates both apoptosis and autophagy through the c-Jun/ATG16L2 axis, and pharmacological inhibition of TRAF6 effectively suppresses tumor growth [8]. Notably, TRAF6-mediated ubiquitination processes exhibit diverse oncogenic functions: it drives sphingosine kinase 1 (SPHK1)-dependent autophagy to facilitate colorectal cancer (CRC) metastasis [12], enhances programmed death-ligand 1 (PD-L1) expression in melanoma via YAP1-TFCP2 transcriptional activation [9], and maintains cancer stemness through POU class 5 homeobox 1 (POU5F1)-mediated stabilization in gastric cancer [13]. Furthermore, chronic inflammation, particularly IL-1 signaling, promotes tumor progression by shaping a pro-tumorigenic microenvironment [14]. In squamous cell carcinoma, THG-1 amplifies IL-1 signaling by stabilizing TRAF6, positioning TRAF6 as a crucial mediator of tumor-associated inflammation [15]. Although these research results indicate that TRAF6 is a regulatory factor with multiple functions in cancer, the specific functions of TRAF6 in different tissues and its possible dual roles (promoting and inhibiting tumor growth) in different cancer types still need to be further clarified. Furthermore, the therapeutic significance of targeting TRAF6 for various kinds of malignant tumors warrants further exploration.

In this review, we systematically evaluate the accumulating evidence implicating TRAF6 in cancers, with particular emphasis on elucidating its fundamental molecular mechanisms. Our analysis focuses on three key aspects: TRAF6-mediated regulation of autophagy, its integration of inflammatory signaling pathways, and its critical contributions to tumor microenvironment (TME). We further summarized how TRAF6-regulated substrates modulate inflammatory responses and tumor progression. We also critically assess current therapeutic strategies targeting TRAF6, including the development of small-molecule inhibitors, emerging natural compounds, and emerging *miRNA*-based regulatory approaches. Collectively, we analyze TRAF6 as a multifaceted regulator in cancer biology and highlight its promising potential as a therapeutic target.

## 2. Structure and Function of TRAF6

**Figure 1:**
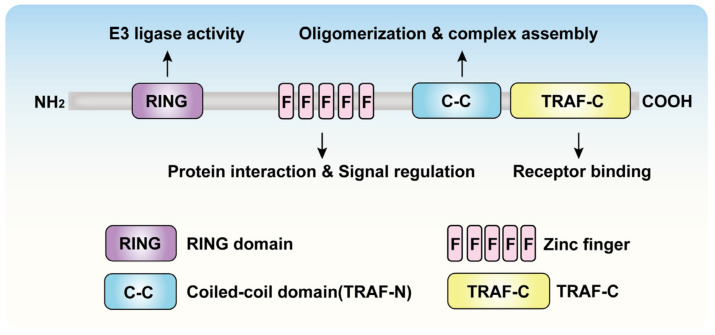
Schematic diagram of the domain architecture of TRAF6. TRAF6 contains a RING finger domain, Zinc finger domain, coiled-coil domain, and TRAF-C domain, which are sequentially distributed from the N-terminus to the C-terminus and perform distinct biological functions.

TRAF6 is a 522-amino acid protein (~59.4 kDa) [16,17]. Structural studies established its individual crystal structure and its complex structures with peptides derived from TNFR superfamily members CD40 and TRANCE-R [16]. These findings provided foundational insights into TRAF6-mediated signal transduction mechanisms. TRAF6 protein comprises distinct functional domains: an N-terminal RING-type zinc finger domain (RING domain), followed by multiple zinc fingers, a coiled-coil domain (CC domain), and a C-terminal TRAF-C domain (Figure 1) [18]. The N-terminal region specifically encompasses the RING domain, a linker helix, and four zinc finger motifs [19]. The RING domain catalyzes the formation of K63-linked polyubiquitin chains by binding to E2 ubiquitin-conjugating enzymes (such as the Ubc13/Uev1A complex) [20]. This domain plays a crucial role in the NF-κB signaling pathway by regulating the activity of transforming growth factor-β-activated kinase 1 (TAK1) to activate downstream signals [21,22]. Notably, TRAF6 exhibits both homodimerization and heterodimerization capabilities with other TRAF proteins. Mechanistically, the RING domain mediates polyubiquitination, a key step regulating E2 enzyme activity [23]. Activation of the E2∼Ub thioester conjugate requires cooperative engagement with both monomers of a TRAF6 dimer, wherein the RING domain of one monomer binds the E2 enzyme while conjugated ubiquitin interacts with adjacent structural elements [24]. The multiple ZZ domains of TRAF6 support the full functionality of the RING finger domain, such as maintaining the spatial conformation of the RING structure to ensure the catalytic efficiency of the E3 ubiquitin ligase [24].

The CC domain is positioned in the central region of TRAF6 and is essential for oligomerization, enabling the formation of functional complexes that facilitate efficient signal transduction [25,26]. Oligomerization enhances TRAF6’s ubiquitin chain transfer activity, particularly through K63-linked polyubiquitination, which propagates signaling cascades such as NF-κB activation [27]. Additionally, the C-terminus of the TRAF-C domain serves as a critical platform for interactions with downstream signaling proteins and receptor molecules, including those involved in immune responses [28]. Moreover, TRAF6 domains function cooperatively. For instance, the RING and ZZ domains work together to regulate ubiquitination, activating NF-κB and MAPK signaling pathways and thereby triggering inflammatory responses [29]. The CC and TRAF-C domains collaborate to ensure efficient complex assembly and signal transmission stability, promoting precise activation of signaling molecules [25]. Therefore, TRAF6 mediates complex signal transduction through its RING finger, zinc finger, helix-helix, and TRAF-C domains. By targeting and inhibiting the E3 ubiquitin ligase activity of the RING finger domain, it may suppress over-activated inflammatory signaling pathways and has the potential for treating inflammation and related cancers.

## 3. Substrates of TRAF6

As an E3 ubiquitin ligase, TRAF6 primarily catalyzes K63- and K48-linked polyubiquitination to regulate the stability and activity of diverse substrate proteins [1,30]. These modifications enable TRAF6 to play pivotal roles in tumorigenesis and disease progression. Here, we summarize TRAF6 substrates and elucidate how their ubiquitination drives tumor initiation and progression (Table 1).

### 3.1. TAK1

TAK1 is an important member of the MAP3K family [79]. It is responsible for upstream signal transduction and activates the downstream MAP2K-MAPK cascade, thereby regulating cell proliferation, differentiation, apoptosis, and inflammatory responses [80,81]. TRAF6 mediates the K63 ubiquitination of TAK1, a crucial regulatory step in TAK1 activation [82]. In primary membranous nephropathy, TRAF6 directly interacts with and ubiquitinates TAK1 through K63-linked ubiquitination, thereby activating the GSDMD/Caspase-1 axis and leading to pyroptosis of cells [83]. Furthermore, the recruitment of TAB1 (TAK1-binding protein 1) by proteins such as repulsive guidance molecule BMP co-receptor b promotes the formation of the TRAF6-TAB1-TAK1 complex, which is essential for TRAF6-mediated K63-linked ubiquitylation and subsequent phosphorylation of TAK1 [84]. In gastric cancer cells exhibiting resistance to 5-fluorouracil (5-FU), TRAF6 upregulation facilitates nuclear translocation of the NF-κB subunit p65, while TRAF6 downregulation suppresses this process [7]. Moreover, the African Swine Fever Virus protein I177L promotes the ubiquitination of TRAF6, thereby enhancing the binding of TRAF6 to TAK1. This further induces the ubiquitination and phosphorylation of TAK1, ultimately activating the NF-κB signaling pathway and driving the inflammatory response [85]. Therefore, TRAF6 activates the NF-κB and MAPK pathways by TAK1, thereby promoting inflammation, immune responses, and tumorigenesis.

### 3.2. Beclin1

Beclin1, also known as BECN1, is a key regulatory protein in initiating autophagy [86]. In macrophages, TRAF6 mediates Beclin1 K63-linked ubiquitination at K117, which activates autophagy through TLR4 signaling [87,88]. Furthermore, the ubiquitination of Beclin1 at the K117 site mediated by TRAF6 is essential for the osteoclast differentiation stimulated by receptor activator of nuclear factor-κB ligand (RANKL) [33]. AMP-activated protein kinase alpha 1 enhances the ubiquitination of BECN1 through TRAF6, promoting the induction of autophagy and the development of cancer [89]. Conversely, CRBN inhibits the TRAF6-induced ubiquitination of Beclin1, thereby suppressing the autophagy activation triggered by TLR4 stimulation [32]. Moreover, the deubiquitinating enzyme A20 directly binds to Beclin1, reversing the TRAF6-mediated ubiquitination modification of Beclin1 [87,88]. In lung cancer, USP15 reverses the TRAF6-mediated ubiquitination of Beclin1, thereby inhibiting autophagy and negatively regulating cell migration and invasion induced by TLR4 stimulation [90]. Therefore, TRAF6 mediates ubiquitination of Beclin1 plays a crucial role in autophagy and tumor migration and invasion triggered by TLR4 stimulation.

### 3.3. AKT

AKT kinase is central to cellular homeostasis, governing diverse processes such as glycogen metabolism and tumorigenesis, and its dysregulation contributes to diseases including cancer and insulin resistance [91,92]. Ubiquitination serves as a critical post-translational modification for AKT, modulating its kinase activity, membrane translocation, and overall activation dynamics [93]. TRAF6-induced ubiquitination of AKT represents a key non-classical pathway for AKT activation [93]. Specifically, TRAF6 promotes AKT translocation to the plasma membrane by catalyzing ubiquitination at lysine residues K8 and K14, thereby regulating the phosphatidylinositol 3-kinase (PI3K)/AKT signaling pathway [94,95]. Upon insulin stimulation, the activated insulin receptor (IR) enhances TRAF6-mediated ubiquitination of AKT1 at K8 and K14 residues [34]. In cancer, TRAF6-mediated AKT ubiquitination facilitates disease progression. For example, in triple-negative breast cancer (TNBC), U5F1-mediated inhibition of K63-linked ubiquitination of TRAF6, which is essential for TRAF6 activation and its downstream signaling transduction, activates AKT signaling, synergizing with TRAF6 to promote oncogenesis [96]. OTUB2 further amplifies this axis by directly activating the TRAF6/AKT pathway to drive TNBC malignancy [97]. Furthermore, the endoplasmic reticulum membrane protein derlin 1 interacts with TRAF6, enhancing AKT ubiquitination and influencing breast cancer development through downstream pathway regulation [98]. In non-small cell lung cancer (NSCLC), TRAF6 ubiquitinates and activates AKT, thereby enhancing HIF-1-mediated hexokinase-2 transcription and strengthening glycolysis [99]. Moreover, lysine demethylase 4B (KDM4B) promotes TRAF6-mediated AKT ubiquitination and activation, thereby facilitating glucose metabolism and growth in CRC [100]. These findings highlight that TRAF6 orchestrates ubiquitination events that potentiate AKT activity through ubiquitin transfer.

### 3.4. MST1/STK4

Mammalian Ste20-like kinase 1/serine/threonine kinase 4 (MST1/STK4) is a core component of the Hippo signaling pathway, which regulates critical processes including cell proliferation, differentiation, and tumor suppression [101]. TRAF6 directly interacts with and ubiquitinates MST1, primarily through K63-linked polyubiquitination. For instance, TRAF6 mediates the LPS-induced activation of MST1 through K63-linked polyubiquitination, which causes negative feedback regulation of MST1 on the LPS-induced pathway and leads to the production of cytokines in macrophages [35]. Conversely, in pancreatic cancer, TRAF6 promotes the migration and colony formation of tumor cells by promoting the ubiquitination and degradation of MST1 [36]. Notably, USP46 counteracts this process in hepatocellular carcinoma (HCC) by removing the TRAF6-mediated K48-linked ubiquitination from MST1, thereby suppressing tumor progression [102]. Collectively, the TRAF6-MST1 axis demonstrates context-dependent regulation of Hippo signaling through antagonistic ubiquitination mechanisms.

### 3.5. IRF

The interferon regulatory factor (IRF) family consists of nine major members (IRF1-IRF9), which are critical for regulating interferon expression and innate immune responses. TRAF6 plays a pivotal role in post-translationally modifying IRF proteins through site-specific ubiquitination to precisely regulate their stability and activity. Specifically, TRAF6 catalyzes Lys70 ubiquitination of IRF3 through K48-linked ubiquitination, leading to the degradation of the IRF3 protein and terminating its transcriptional activity. In 5-FU-resistant gastric cancer cells, TRAF6-induced IRF3 degradation promotes the translocation of the NF-κB-p65 subunit to the nucleus, thereby enhancing tumor cell proliferation [7]. Conversely, TRAF6-mediated K63-linked ubiquitination regulates IRF5 and IRF7 activation without inducing degradation, thereby inhibiting the innate immune response and promoting viral immune escape [37,38,39]. Further research indicates that the region between IRF-7 amino acids 238 and 285 is crucial for the binding and ubiquitination of TRAF6 to IRF-7, which is essential for the activation of IRF-7 and subsequent type I interferon (IFN-α) production in innate immune signaling [38]. Therefore, TRAF6 exerts differential ubiquitination modifications to precisely regulate the stability and activity of proteins such as IRF3, IRF5, and IRF7, thereby influencing the development of immune cells, inflammatory responses, and antiviral immune responses.

### 3.6. STAT

The signal transducer and activator of transcription (STAT) family is a class of signal transducers and transcriptional activators [103]. STAT3 serves as a crucial mediator of cellular responses to cytokines such as IL-6 and IL-10, and growth factors [104]. TRAF6 directly regulates STAT3 transcriptional activity via ubiquitination, thereby negatively regulating the JAK-STAT signaling [42]. Additionally, STAT3 provides negative feedback regulation of the TRAF6 signaling pathway by downregulating Ubc13 expression [105]. Research indicates that TRAF6 enhances the immunosuppressive function of myeloid-derived suppressor cells (MDSCs) by mediating K63-linked ubiquitination of STAT3 [40]. TRAF6-mediated K63-linked ubiquitination of STAT3 promotes its membrane recruitment and subsequent phosphorylation in response to bacterial infections [41]. Moreover, TRAF6 binds to STAT6 and inhibits K48-linked ubiquitination, stabilizing STAT6 and playing a critical role in TLR-triggered M1 macrophage activation [43]. Consequently, TRAF6 acts as a pivotal regulator in STAT signaling pathways, significantly influencing cell growth, immune responses, and cancer progression.

### 3.7. MYC

The transcription factor MYC is a pivotal oncogene whose dysregulation contributes to the pathogenesis of diverse cancers [106,107]. TRAF6 critically regulates MYC function through ubiquitination, directly modulating its activity or indirectly influencing expression levels. In myeloid leukemia, TRAF6 ubiquitinates MYC, which represses functional activity by antagonizing acetylation modifications without altering protein stability [44]. In HCC, TRAF6 overexpression is associated with poor prognosis and promotes tumor growth, while TRAF6 induces k63-linked ubiquitination of HDAC3, thereby disrupting the interaction between HDAC3 and c-Myc, and promoting the stability of c-Myc protein [45]. These regulatory mechanism highlights the complex role of TRAF6 in the intracellular signaling network.

## 4. TRAF6 Is Abnormally Expressed and Is Associated with Poor Prognosis

TRAF6 is frequently abnormally highly expressed in various types of malignant tumors, leading to enhanced tumor cell proliferation, resistance to apoptosis, increased invasiveness, and metastatic potential [108]. In acute myeloid leukemia (AML), TRAF6 is significantly upregulated compared to healthy controls, with studies showing markedly elevated mRNA and protein levels in AML cell lines and patient samples [109]. Similarly, in solid tumors such as HCC, GBM, and lung adenocarcinoma (LUAD), TRAF6 overexpression correlates with aggressive disease phenotypes and poor clinical outcomes. In HCC, TRAF6 is overexpressed and its knockdown dramatically attenuates tumor cell growth, suggesting it may represent a potential therapeutic target [45]. TRAF6 activates the Wnt/β-catenin pathway via LEF1-mediated transcriptional enhancement, promoting epithelial–mesenchymal transition (EMT) and facilitating tumor invasion and metastasis [110]. Similarly, the expression of TRAF6 in the esophageal cancer tissues was significantly increased, which was also related to the patient’s age and the shortened recurrence time of the tumor [111]. In gastric cancer, TRAF6 upregulation contributes to tumor aggressiveness, chemoresistance, and poor prognosis, with suppression of TRAF6 impairing proliferation and tumor growth [112,113]. In pancreatic cancer, the expression of TRAF6 is upregulated in pancreatic cancer tissues, promoting cell proliferation and migration [114]. Conversely, the downregulation of TRAF6 weakens the tumorigenicity of pancreatic cancer cells both in vitro and in vivo [114]. In breast cancer, TRAF6 is generally highly expressed, especially in TNBC, suggesting its utility as a metastatic biomarker [115].

**Figure 2:**
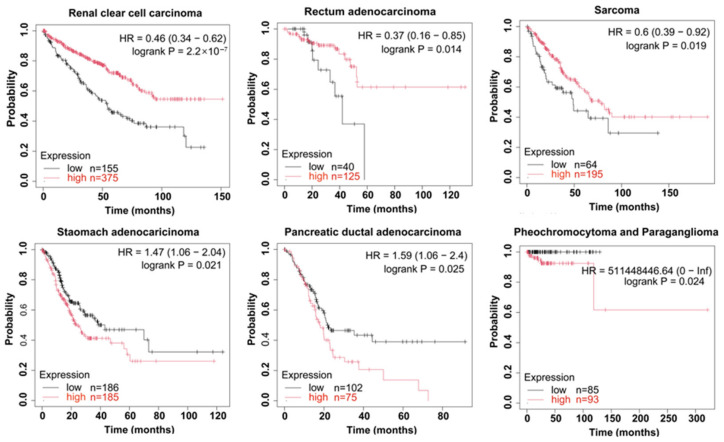
TRAF6 expression is associated with overall survival in many human cancers. TRAF6 plays a dual role in cancer patients: on the one hand, high expression of TRAF6 promotes the death of cancer patients, such as gastric adenocarcinoma (*p* = 0.021), pancreatic ductal adenocarcinoma (*p* = 0.025), and pheochromocytoma and paraganglioma (*p* = 0.024); on the other hand, high expression of TRAF6 inhibits the death of cancer patients, such as in renal clear cell carcinoma (*p* = 2.2 × 10^−7^), rectal adenocarcinoma (*p* = 0.014), and sarcoma (*p* = 0.019).

Notably, accumulating evidence indicates that TRAF6 exerts dichotomous roles in cancer progression and prognosis, with its functional outcome exhibiting strict cancer type-specificity. For instance, elevated TRAF6 is positively correlated with advanced N stage and pathological stage, and the prognosis of patients in the positive group was significantly worse than that of patients in the negative group [116,117]. In CRC patients (*n* = 100), TRAF6 expression in the N1 and N2 stages was higher than that in the N0 stage [118]. Moreover, elevated TRAF6 expression correlates with bone metastasis, leading to a significantly lower 5-year survival rate [119]. Furthermore, in lung cancer patients, the overexpression of the *TRAF6* gene was significantly correlated with the response to platinum-based chemotherapy (*p* = 0.039), stage (*p* = 0.043), and PS score (*p* = 0.045) [120]. In lung cancer cases (*n* = 365), TRAF6 overexpression was related to the clinical tumor-node-metastasis stage, tumor size, and lymph node metastasis status [121,122]. GBM studies reveal that TRAF6 expression significantly correlates with matrix metalloproteinase 9 levels and serves as an independent prognostic factor [123]. Urothelial bladder cancer analyses (*n* = 126) identify high TRAF6 expression as an independent predictor of poor prognosis (HR = 1.23, *p* = 0.037) and increased recurrence (HR = 1.24, *p* = 0.011) [124]. Moreover, pan-cancer analysis reveals that TRAF6 expression correlates with improved overall survival (OS) in patients with gastric adenocarcinoma (*p* = 0.021), pancreatic ductal adenocarcinoma (*p* = 0.025), and pheochromocytoma and paraganglioma (*p* = 0.024) (Figure 2). On the other hand, TRAF6 exhibits tumor-suppressive functions in specific cancer contexts, where high TRAF6 expression correlates with favorable prognosis. Notably, TRAF6 exerts a critical tumor-suppressive function in myeloid malignancies, particularly in AML [44]. In CRC, its role remains controversial: TRAF6 promotes selective autophagic degradation of β-catenin, thereby inhibiting Wnt/β-catenin signaling and cancer cell metastasis [46], while others demonstrate that TRAF6 also facilitates tumor cell survival and progression by inhibiting RIPK1/RIPK3/MLKL-mediated necroptosis [50]. Furthermore, pan-cancer analysis reveals that TRAF6 expression correlates with improved OS in patients with clear cell renal cell carcinoma (*p* = 2.2 × 10^−7^), rectal adenocarcinoma (*p* = 0.014), and sarcoma (*p* = 0.019) (Figure 2). According to these reports, data from the Kaplan–Meier plotter (http://kmplot.com) indicate that dysregulation of TRAF6 is associated with the OS rates of various human cancers.

## 5. The Core Mechanism of TRAF6 Regulation in Cancer

### 5.1. TRAF6 Regulates Autophagy

**Figure 3:**
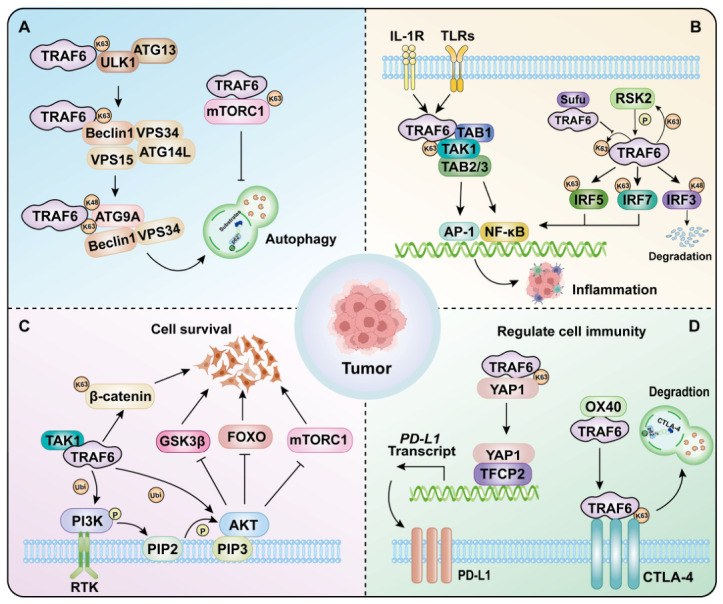
The core regulatory mechanism of TRAF6 in cancer. (**A**) TRAF6 regulates autopha gy and proliferation in tumor cells by ubiquitinating key autophagy-related factors, such as ULK1, Beclin1, and Atg9. (**B**) TRAF6 regulates inflammatory responses via autoubiquitination and ubiquitinating TAK1, IRF3/5/7, thereby influencing tumor cell proliferation. (**C**) TRAF6 regulates tumor cell survival by ubiquitinating factors such as β-catenin, PI3K, and AKT. (**D**) TRAF6 modulates tumor immune responses via ubiquitination of YAP and CTLA-4.

TRAF6 plays a pivotal role in the regulation of autophagy in tumor cells (Figure 3A) [125]. In melanoma cells, knocking out TRAF6 can simultaneously induce apoptosis and autophagy [8]. Mechanistically, TRAF6 regulates the expression of ATG16L2 through c-Jun, and knockdown of ATG16L2 leads to increased autophagy and apoptosis [8]. The TRAF6 inhibitor Sinicin induces autophagy and apoptosis through the TRAF6/c-Jun/ATG16L2 signaling pathway, effectively inhibiting the growth of melanoma cells [8]. Furthermore, TRAF6 mediated K63-linked ubiquitination of BECN1 to stabilize BECN1, thereby activating the AMPK signaling pathway to promote prostate cancer proliferation [89]. Conversely, P62/SQSTM1 competitively inhibits the formation of the TRAF6—BECN1 complex, thereby inhibiting the migration and invasion of cancer cells [126]. TRAF6 also mediates mixed K63/K48-linked ubiquitination of ATG9A, strengthening its association with the Beclin 1-VPS34 complex and activating VPS34 kinase activity, which is essential for oxidative stress-induced autophagy [30]. Furthermore, TRAF6 mediated K63-linked ubiquitination of unc-51-like autophagy activating kinase 1 (ULK1), promoting its stabilization and activation. The granular protein (GCA) activates autophagy through the GCA-TRAF6-ULK1 axis, thereby promoting imatinib resistance in chronic myeloid leukemia [78]. Similarly, AMBRA1 facilitates TRAF6-mediated K63-linked ubiquitination of ULK1, enhancing its stability, oligomerization, and functional activation [127]. In gastric cancer, TRAF6 promotes tumor cell viability, migration, and proliferation via autophagy induction, with these effects being reversible upon autophagy inhibition [112]. These findings collectively position TRAF6 as a central regulator of autophagy across various cancers and exhibit tissue-specific effects.

### 5.2. TRAF6 Regulates the Inflammatory Signal

TRAF6 functions as a critical signaling hub in inflammatory pathways, integrating signals from TLRs and IL-1Rs to activate the NF-κB pathway (Figure 3B) [128]. Dysregulation of TRAF6-mediated signaling is strongly associated with inflammatory disorders and tumorigenesis, as evidenced by its overexpression in cancers such as HCC and gastric cancer [7,129]. Ribosomal S6 kinase 2 (RSK2) phosphorylates TRAF6 to mediate LPS-induced inflammatory signaling, while Sufu restricts the pulmonary inflammation caused by sepsis by regulating the K63-linked ubiquitination of TRAF6 [130,131]. TRAF6-driven NF-κB activation promotes excessive production of pro-inflammatory cytokines, such as TNF-α and IL-6, contributing to a chronic inflammatory state that enhances tumor cell proliferation and survival via autocrine and paracrine mechanisms [80]. In microglia, the TRAF6/NF-κB axis modulates neuroinflammation through *miRNA*-dependent pathways, including *miR-146b-5p* [132]. Notably, *TRAF6* knockdown using shRNA significantly attenuates proliferation and tumor growth in 5-FU-resistant gastric cancer models by inhibiting nuclear translocation of NF-κB-p65 [7]. Furthermore, TRAF6’s regulatory role extends to organ-specific inflammation and injury. For instance, TRAF6 promotes K6-linked multimerization and activation of apoptosis signal-regulating kinase 1 (ASK1), triggering the release of pro-inflammatory and pro-fibrotic factors from hepatocytes. Genetic evidence shows that Traf6+/− mice exhibit attenuated liver inflammation and fibrosis, while TRAF6 overexpression exacerbates these pathological changes [73]. These findings highlight TRAF6 as a pivotal therapeutic target for mitigating inflammation-driven pathologies.

### 5.3. TRAF6 Regulates Survival-Promoting Signal

TRAF6 promotes tumor cell proliferation and survival through multiple mechanisms. TRAF6 regulates the PI3K-AKT-GSK3β cascade, which is essential for TNF-mediated cell survival (Figure 3C) [133,134]. Mechanistically, TRAF6 potentiates PI3K-AKT signaling via dual ubiquitination mechanisms: direct K63-linked ubiquitination of AKT, and stabilization of phosphatidylinositol-4,5-bisphosphate 3-kinase catalytic subunit alpha (PIK3CA) via ubiquitination, thereby enhancing lipid kinase activity and downstream oncogenic signaling [34,49]. In GBM, the LSINCT5/*miR-451* axis exploits TRAF6-dependent mechanisms to co-activate PI3K/AKT, Wnt/β-catenin, and NF-κB pathways, collectively driving tumor progression and metastatic dissemination [135]. Moreover, the TRAF6-Wnt/β-catenin axis plays a critical role in tumorigenesis across multiple cancer types. For instance, TRAF6 silencing markedly attenuates Wnt3a-induced β-catenin accumulation and downstream target gene expression in PC3U and SW480 cancer cells [136]. In LUAD, basic leucine zipper and W2 domains 2 (BZW2) enhance TRAF6-mediated ubiquitination and degradation of GSK3β, leading to Wnt/β-catenin pathway activation that drives tumor progression and metastasis [75]. Combinatorial inhibition of TRAF6 and TXNIP in lung cancer models upregulates E-cadherin while suppressing invasive and migratory capacities [53]. In addition, TRAF6 plays a pivotal role in stem cell homeostasis. In skeletal muscle, TRAF6 inhibition enhances satellite cell activation and muscle regeneration through Notch signaling upregulation [137,138]. These findings collectively position TRAF6 as a master regulator of multiple oncogenic pathways and a potential therapeutic target across diverse malignancies.

### 5.4. TRAF6 Regulates Tumor Immune

TRAF6 critically regulates tumor immunity through multiple mechanisms. Specifically, TRAF6 stabilizes YAP1 via K63-linked ubiquitination, promoting the formation of the YAP1/TFCP2 transcriptional complex and subsequent upregulation of PD-L1 transcription (Figure 3D). Notably, bortezomib, a classic proteasome inhibitor, can suppress the E3 ligase activity of TRAF6, thereby decreasing PD-L1 membrane expression and potentiating CD8+ T cell-mediated cytotoxicity against melanoma [9]. Additionally, TRAF6 enhances the immunosuppressive function of MDSCs in a RING domain-dependent manner, thereby suppressing CD8+ T cell-mediated antitumor immunity. TRAF6 and arginase 1 are highly expressed in MDSCs, confirming their role in MDSC-mediated immune suppression [40]. Furthermore, TRAF6 facilitates CTLA-4 degradation via the OX40-TRAF6 axis, augmenting T cell antitumor responses; this mechanism is validated in tumor-bearing mice and cancer patients, suggesting therapeutic potential for improving T cell-based immunotherapy [76]. Overall, TRAF6 orchestrates central functions in the tumor immune microenvironment, including MDSC activity, T cell activation, and immune checkpoint modulation.

## 6. Therapeutic Strategies Targeting TRAF6

**Figure 4:**
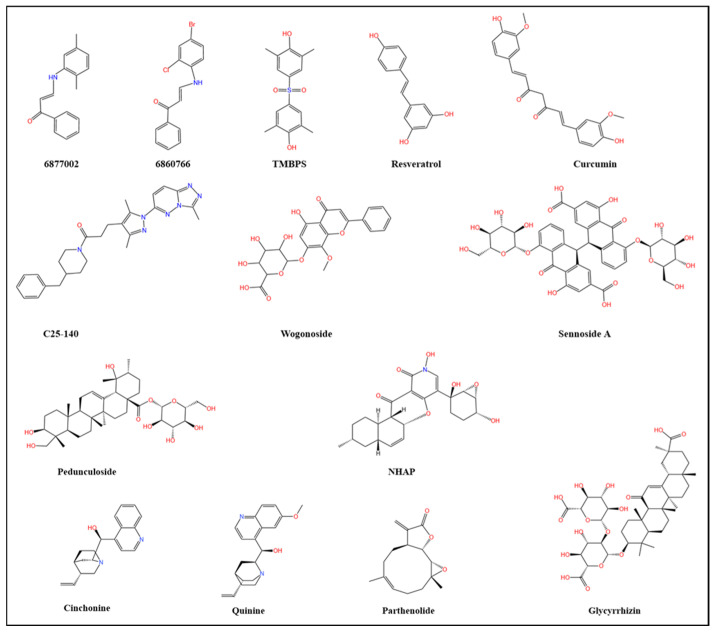
Representative compounds of TRAF6 inhibitors. TRAF6 inhibitors mainly include small molecule inhibitors such as 6877002, 6860776, and C25-140, as well as natural products such as curumin, resveratrol, and Sennoside A. These inhibitors effectively target TRAF6 and inhibit the TRAF6-dependent signal cascade.

**Figure 5:**
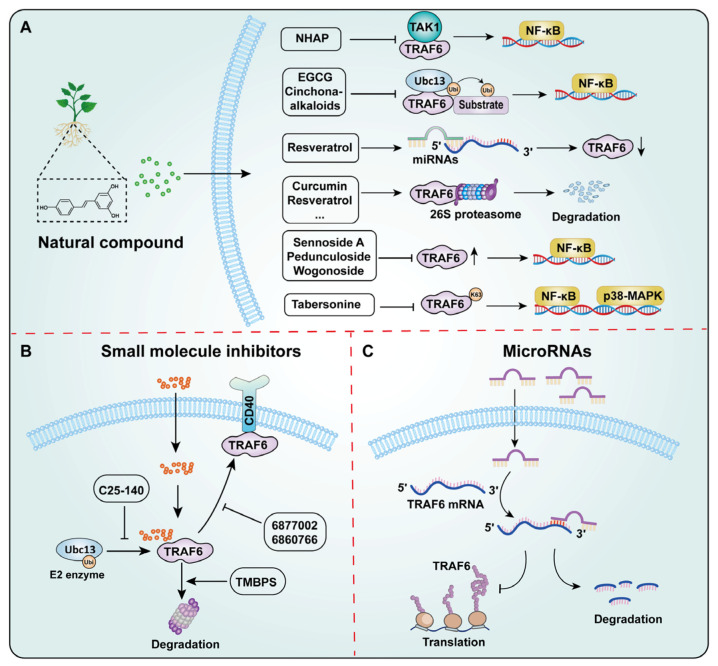
Schematic of TRAF6 Inhibitor Mechanism of Action. (**A**) Natural compounds suppress the function of TRAF6 by promoting its degradation, inhibiting its expression, and attenuating its enzymatic activity. (**B**) Small-molecule inhibitors inhibit the function of TRAF6 by promoting its degradation and blocking the binding of TRAF6 to the E2 enzymes Ubc13 and CD40. (**C**) MicroRNAs inhibit TRAF6 expression via targeting its 3′ untranslated region, suppressing tumorigenesis in most tumors while promoting it in a few.

Multiple pharmacological strategies have been developed to suppress TRAF6-dependent signaling cascades. Direct intervention employs small-molecule inhibitors to selectively abrogate TRAF6 function, whereas indirect approaches focus on modulating TRAF6-dependent upstream regulators or downstream effectors (Table 2) (Figure 4 and Figure 5).

### 6.1. Natural Compounds Targeting TRAF6

Natural compounds have become ideal candidates for the prevention and treatment of diseases such as cancer due to their wide sources, low cost, and high biocompatibility [139]. They have also been proven to target TRAF6 and regulate its function (Figure 5A). These natural compounds have the advantages of low toxicity and good biocompatibility, making them promising candidates for cancer treatment.

#### 6.1.1. Curcumin

Curcumin, a natural polyphenolic compound, demonstrates broad-spectrum anti-cancer activity through multiple mechanisms, including modulation of signaling pathways, TME regulation, apoptosis induction, and tumor progression inhibition [140]. The compound exhibits potent anti-inflammatory properties through distinct mechanisms. In mammary gland epithelial cells, curcumin suppresses TRAF6 expression and inflammatory factor production, showing synergistic effects with ciprofloxacin in preventing Staphylococcus aureus-induced mastitis [141]. Furthermore, the curcumin-6-shogaol-10-shogaol combination demonstrates superior efficacy in reducing pro-inflammatory mediators in LPS/IFN-γ-stimulated macrophages compared to individual components [142]. This effect is mediated through downregulation of the TLR4/TRAF6/MAPK pathway and inhibition of NF-κB nuclear translocation, independent of Nrf2 activation [142]. Neuroprotective effects of curcumin involve restoration of oxidant/antioxidant balance and suppression of the TLR4/MyD88/TRAF6/NF-κB axis, subsequently reducing pro-inflammatory cytokines TNF-α and IL-1β [143]. curcumin also modulates autophagic flux, potentially restoring autophagy homeostasis through TRAF6-dependent pathways [144,145]. Genetic knockout experiments provide definitive evidence that curcumin’s regulatory effects on both osteoclastogenesis and autophagy are strictly TRAF6-dependent-the compound loses its bioactivity in TRAF6-deficient systems [144]. Specifically, curcumin inhibits osteoclast differentiation by inducing TRAF6 degradation, while simultaneously regulating autophagy-related pathways to reestablish autophagic balance. Despite these promising mechanistic findings, curcumin’s clinical translation remains constrained by poor bioavailability and suboptimal pharmacokinetic properties.

#### 6.1.2. Resveratrol

Resveratrol is a naturally occurring polyphenol that has been extensively studied for its therapeutic potential in the case of cancer, cardiovascular disease, and diabetes [146,147]. Research indicates that TRAF6 is an important target for Resveratrol to inhibit TLR4-mediated NF-κB activation, which is crucial in anti-cancer, anti-inflammatory, and antioxidant effects [147,148]. For instance, resveratrol alleviates visceral pain caused by colitis by inhibiting the TRAF6/NF-κB signaling pathway in the spinal cord [149]. Furthermore, resveratrol inhibits LPS-induced inflammatory damage in mouse microglia by regulating the *miR-146a-5p*/TRAF6/NF-κB axis [150]. In cancer, resveratrol can disrupt CRC metastasis by regulating the *miR-125b-5p/TRAF6* signaling axis [151]. Moreover, resveratrol inhibits prostate cancer cell proliferation and migration by mediating the degradation of TRAF6 [152]. These results indicate that the TRAF6/NF-κB signaling pathway is the key mechanism by which resveratrol exerts its anti-cancer, anti-inflammatory, and antioxidant effects.

#### 6.1.3. Sennoside A

Sennoside A (SA) is a major component of rhubarb that has been proven to be effective in treating various diseases [153]. For instance, SA upregulates the expression of suppressor of cytokine signaling 1 (SOCS1) by inhibiting DNA methyltransferase 1-mediated methylation of the SOCS1 promoter, thereby inhibiting the proliferation of HSCs, reducing the expression of α-SMA/Col1α1 and the inflammatory response, and exerting an anti-liver fibrosis effect [154]. In prostate cancer, SA regulates cell proliferation and autophagy by inhibiting the PI3K/AKT/mTOR axis in vitro and in vivo [155]. In non-small cell lung cancer, Sennoside A inhibits cancer cell proliferation, invasion, and TME by inactivating the TRAF6/NF-κB pathway [156]. Furthermore, SA inhibits the TRAF6/NF-κB pathway, thereby alleviating neuroinflammation, ferroptosis, and oxidative stress in AD mice and lipopolysaccharide-induced BV2 cells, and improving cognitive function [157]. Therefore, SA inhibits the NF-κB signaling pathway, exerting therapeutic effects on liver fibrosis, cancer, and neurodegenerative disorders by alleviating inflammation and promoting cellular protection.

#### 6.1.4. Pedunculoside

Pedunculoside (PE) is a monomeric compound chemically extracted from the traditional medicine of wintergreen bark [158]. It has potential pharmacological effects, including antibacterial, anticancer, anti-inflammatory, anti-anxiety, and local anesthetic properties [159,160]. For instance, PE inhibits EMT by regulating the MAPK and Nrf2 pathways, reverses the expression of EMT-related proteins induced by TGF-β1, and inhibits NSCLC cell migration and invasion [160]. Moreover, it can also restore the sensitivity of A549/GR cells to gefitinib and significantly inhibit lung metastasis in mouse models, indicating that it is a therapeutic agent for inhibiting the metastasis of NSCLC and improving the potential of gefitinib resistance [160]. In bladder cancer, PE significantly reduces the viability, invasion ability, and tumor growth of bladder cancer cells by inhibiting the TRAF6/NF-κB signaling pathway [161]. The mechanism includes down-regulating the expression of TRAF6 protein, reducing NF-κB activation, and decreasing the pro-tumor effects induced by M2-type macrophages (such as inhibiting markers like Ki-67 and Vimentin). Animal experiments further confirmed that PE inhibits tumor growth through this pathway [161].

#### 6.1.5. Wogonoside

Wogonoside is a natural flavonoid glucuronide primarily derived from *Scutellaria baicalensis Georgi*, which has demonstrated potent anti-inflammatory properties by regulating key signaling pathways such as NF-κB and MAPKs [162,163]. For instance, Wogonoside significantly alleviated ovalbumen-induced inflammatory cell infiltration, mucus secretion, and goblet cell hyperplasia, reduced the expression of Th2 cytokines (IL-4, IL-5, IL-13) and mucoproteins (MUC5AC and MUC5B), and improved lung function by inhibiting the activation of the NF-κB/STAT6 pathway [164]. In pancreatic cancer, Wogonoside inhibits the TRAF6/NF-κB pathway to downregulate the expression of oncogenic factors such as TRAF6, VCAM1, CD44, and MMP14, thereby inhibiting the proliferation of pancreatic cancer cells, promoting apoptosis, and blocking the stem cell-like transformation and mesenchymal transformation in the TME [165]. Furthermore, Wogonoside inhibits the proliferation and metastasis in prostate cancer, liver cancer, and endometrial cancer by regulating PI3K/Akt, Hippo signaling, and Wnt/beta-catenin signaling [166,167,168].

#### 6.1.6. *Cinchona* alkaloids

*Cinchona* alkaloids, derived from the bark of *Cinchona* trees, are a group of naturally occurring compounds primarily recognized for their pharmacological properties in treating malaria [169]. These alkaloids include four major compounds: quinine, quinidine, cinchonine, and cinchonidine [169]. Recent studies suggest that *Cinchona* alkaloids have promising therapeutic pharmacological activities, such as anti-inflammatory, antioxidant, antiarrhythmic, and anticancer properties [170]. Among them, quinine and cinchonine can affect the occurrence and development of tumors by targeting and regulating TRAF6. For instance, quinine exhibits significant anti-proliferative and pro-apoptotic effects in HeLa and A549 tumor cell lines by targeting TRAF6 to inhibit the activation of AKT and inhibiting BCL-2 [171]. Cinchonine can induce apoptosis of HeLa and A549 tumor cells by targeting TRAF6 [172]. Mechanistically, Cinchonine specifically binds to the RING domain of TRAF6, disrupting the interaction between TRAF6 and Ubc13 and thereby inhibiting the AKT and TAK1 signaling pathways [172]. Furthermore, Cinchonine can induce autophagy and apoptosis through the TRAF6/c-Jun/ATG16L2 signaling pathway, thereby inhibiting the growth of melanoma cells [8]. These results indicate that TRAF6 plays a crucial role in the regulation of tumor occurrence and development by *Cinchona* alkaloids.

#### 6.1.7. Parthenolide

Parthenolide is a sesquiterpene lactone derived from the medicinal plant feverfew (*Tanacetum parthenium*), which has shown potent anti-cancer activities and anti-inflammatory properties [173]. The mechanism by which parthenolide exerts its anti-inflammatory and anticancer effects often involves the inhibition of TRAF6-mediated signaling. It was reported that parthenolide can directly or indirectly interfere with TRAF6 activation or its downstream targets, thereby attenuating inflammatory responses and suppressing tumor growth [174,175]. In multiple myeloma (MM), Parthenolide directly binds to and targets TRAF6, thereby inhibiting the activation of the NF-κB signaling pathway in RPMI 8226 cells. This leads to a decrease in the ubiquitinated Nemo level, an increase in -α expression, a reduction in the nuclear content of p65, and inhibition of cell proliferation [176]. This further expands the application value of TRAF6 inhibitors in the treatment of hematological tumors.

#### 6.1.8. Glycyrrhizin

Glycyrrhizin is one of the main active components of the root of licorice. It exerts its pharmacological effects mainly by inhibiting high mobility group protein B1 (HMGB1), and plays a crucial role in inflammatory responses, oxidative stress, and disease progression [177]. For instance, glycyrrhizin regulates the ubiquitination and degradation of TRAF6 by modulating HMGB1, thereby inhibiting the production of inflammatory factors and the M1 polarization of macrophages, and alleviating inflammatory pain [178]. However, the pharmacokinetics and targeted delivery efficiency of glycyrrhizic acid in tumor treatment are insufficient, which limits its clinical application [179].

#### 6.1.9. Natural Alkaloid (-)-N-Hydroxyapiosporamide

(-)-N-Hydroxyapiosporamide (NHAP) is a natural alkaloid compound that exhibits antifungal activity against *Candida albicans* or *Aspergillus fumigatus*, as well as anti-tumor activity [180,181]. In CRC, NHAP can inhibit the NF-κB signaling pathway by targeting the TAK1-TRAF6 complex, thereby inducing apoptosis and autophagic cell death in vitro and in vivo [180]. This study first clarified the anti-tumor mechanism of NHA, laying the foundation for its development as a lead compound for CRC treatment.

### 6.2. Targeting TRAF6 of Small Molecule Inhibitors

#### 6.2.1. TRAF-STOP Inhibitor

TRAF-STOP inhibitors 6877002 and 6860766 can selectively block the interaction between CD40 and TRAF6, thereby inhibiting downstream signaling cascades (Figure 5B) [182]. The inhibitor 6877002 exerts an anti-atherosclerotic effect by blocking the CD40-TRAF6 signaling pathway, inhibiting classical monocyte activation, leukocyte recruitment, and macrophage activation and migration [183]. Preclinical studies demonstrate that co-administration of 6877002 with docetaxel in murine models of breast cancer yields superior therapeutic outcomes compared to monotherapy [184]. This combinatorial approach achieves dual therapeutic effects by simultaneously targeting tumor proliferation and bone metastasis through TRAF6/NF-κB pathway inhibition, offering novel therapeutic potential for advanced breast cancer treatment. These findings indicate that 6877002 has potential for the treatment of breast cancer and other inflammation-related cancers.

#### 6.2.2. TMBPS

Bis (4-hydroxy-3,5-dimethylphenyl) sulfone (TMBPS) is a novel inhibitor that can directly bind to TRAF6, thereby downregulating the expression of TRAF6 (Figure 5B) [129]. In HCC, TMBPS directly bind to TRAF6 and promote its protein degradation, thereby causing HCC cells to stall at the G2/M phase and triggering apoptosis [129]. Mechanistically, TMBPS treatment inhibits AKT/ERK1/2 and p38/MAPK signaling pathways to exert anti-tumor effects [129]. Notably, TMBPS shows a good safety profile, with relatively low toxicity to normal liver cells L0-2 [129].

#### 6.2.3. C25-140

C25-140 is a small-molecule inhibitor that blocks its interaction with the ubiquitin ligase Ubc13, thereby inhibiting the E3 ubiquitin ligase activity of TRAF6 (Figure 5B) [185]. This effect directly inhibits the activation of downstream NF-κB signaling pathways [185]. In macrophages, C25-140 can efficiently inhibit the receptor signals mediated by IL-1β and TNFα, thereby regulating the inflammatory signaling pathways [186]. C25-140 improved the inflammatory and disease outcomes of autoimmune psoriasis and rheumatoid arthritis in the mouse model [185]. Furthermore, C25-140 can inhibit the proliferation of tumor cells by suppressing the activity of TRAF6 and reducing the methylation activity of PRMT5 (Protein Arginine Methyltransferase 5) [77]. When C25-140 is used in combination with PRMT5 inhibitors, it shows a stronger anti-proliferative effect on breast cancer cell lines [77]. These findings suggest that C25-140 has potential application value in TRAF6-overexpressing cancers.

### 6.3. MicroRNAs

MicroRNAs represent a class of endogenous non-coding RNAs that post-transcriptionally regulate gene expression through sequence-specific interactions with target mRNAs [187]. Emerging evidence highlights the critical role of *miRNAs* in regulating TRAF6 expression across various cancer types, with significant implications for tumor progression and therapeutic response (Figure 5C). In breast cancer, the circ-TPGS2/*miR-7*/TRAF6 axis activates NF-κB signaling to promote tumor cell migration, in which circ-TPGS2 overexpression competitively binds *miR-7*, relieving its suppression of TRAF6 [188]. Similarly, *miR-7*-mediated downregulation of *TRAF6* impedes NF-κB activation, thereby suppressing proliferation, migration, and invasion in CRC [189]. Additional CRC studies reveal that *miR-124* directly targets *TRAF6* to inhibit EMT [190], while *miR-125b-5p* suppresses metastasis by attenuating TRAF6-dependent signaling pathways [151]. Furthermore, *miR-140*-mediated downregulation of *TRAF6* impedes the NF-κB/c-Jun pathway, thereby reducing tumor cell proliferation and migration [191]. In osteosarcoma, *miR-124* and *miR-140-3p* function as negative regulators of *TRAF6*, modulating cellular proliferation, apoptosis, and invasive potential [192,193]. Similarly, in prostate cancer, *miR-141-3p* exhibits dual regulatory activity by simultaneously targeting *TRAF5* and *TRAF6* to suppress NF-κB signaling and inhibit bone metastasis [194].

The miR-146 family, particularly *miR-146a*, plays a pivotal role in cancer biology by targeting *TRAF6* to regulate NF-κB signaling. This mechanism influences Th17 cell differentiation and impacts the growth and apoptosis of cervical cancer cells [195,196]. In intestinal epithelial cells, *miR-146a* deficiency promotes CRC development by dysregulating TRAF6-mediated signaling [197]. Furthermore, *miR-146a* exerts tumor-suppressive effects in cervical and liver cancers by inhibiting TRAF6-dependent proliferation and invasion [198]. In pancreatic cancer, *miR-146a-5p* targets the 3′-UTR of *TRAF6* to suppress cell proliferation and enhance gemcitabine chemosensitivity [199]. Conversely, *miR-146b-5p* exhibits pro-tumorigenic activity by inhibiting the expression of TRAF6 in CRC [200], while in glioma, it enhances temozolomide sensitivity by suppressing the TRAF6/AKT/NF-κB axis [201]. Other *miRNAs*, such as *miR-361-3p*, demonstrate therapeutic potential by targeting *TRAF6* to inhibit proliferation and induce apoptosis in MM [202]. Similarly, *miR-429* suppresses HCC cell migration by downregulating TRAF6 and inhibiting NF-κB-mediated p65 nuclear translocation [203]. Additionally, *miR-605-3p* attenuates EMT and metastasis in HCC by targeting *TRAF6* and inhibiting NF-κB signaling [204]. These findings underscore the therapeutic potential of miRNA-mediated TRAF6 regulation as a promising strategy to overcome drug resistance and enhance anti-cancer efficacy. Further investigation into these *miRNA*-*TRAF6* networks may provide novel insights for targeted cancer therapy.

**Table 2:** Inhibitors and microRNA targeting TRAF6 in cancers and inflammatory diseases.

Drug	Mechanism	Disease	References
6877002	Selectively block the interaction between CD40 and TRAF6	Atherosclerosis, breast cancer, inflammation	[182,183,184]
6860766	Selectively block the interaction between CD40 and TRAF6	Inflammation	[182,205]
TMBPS	Binding to TRAF6 and promotes its protein degradation	Hepatocellular carcinoma	[129]
C25-140	Directly binds to TRAF6 and blocks its interaction with Ubc13	Inflammation, breast cancer	[77,185]
Curcumin	Promoting the proteasomal degradation of TRAF6	Inflammation, osteoporosis	[142,145]
Resveratrol	Affecting upstream microRNA, inhibiting TRAF6 expression, and promoting its degradation	Inflammation, prostate cancer, colorectal cancer	[148,149,150,151,152]
Sennoside A	Inhibiting the TRAF6/NF-κB pathway	Non-small cell lung cancer	[156]
Pedunculoside	inhibiting the TRAF6/NF-κB pathway	Bladder cancer	[161]
Wogonoside	Downregulating the expression of TRAF6 and inhibiting the NF-κB signaling pathway	Pancreatic cancer	[165]
*Cinchona* alkaloids	inhibit the interaction between TRAF6 and Ubc13 or specific binding to the RING domain of TRAF6	Cervical cancer, lung cancer	[171,172]
Parthenolide	Directly or indirectly interfering with TRAF6 activation or its downstream targets	Multiple myeloma, inflammation, bacterial-induced osteolytic diseases	[174,175,176]
Glycyrrhizin	Regulating B1 (HMGB1) to regulate the ubiquitination and degradation of TRAF6	Chronic prostatitis	[178]
NHAP	Targeting the TAK1-TRAF6 complex to inhibit NF-κB signaling pathway	Colorectal cancer	[180]
Tabersonine	Reducing K63-linked polyubiquitination of TRAF6	LPS induced acute lung injury	[206]
EGCG	Competitive inhibition of the interaction between Ubc13 and TRAF6	Melanoma	[207]
*miR-7*	Inhibition of TRAF6 expression	Breast cancer, colorectal cancer	[188,189]
*miR-124*	Inhibition of TRAF6 expression	Colorectal cancer, osteosarcoma	[190,192]
*miR-125b-5p*	Reducing TRAF6 expression	Colorectal cancer	[151]
*miR-140*	Targeting the 3′UTR region of *TRAF6* mRNA to reduce TRAF6 protein expression	Colorectal cancer	[191]
*miR-140-3p*	Inhibition of TRAF6 expression	Osteosarcoma	[193]
*miR-141-3p*	Inhibition of TRAF6 and TRAF5 expression	Prostate cancer	[194]
*miR-146a*	Targeting the 3′UTR region of *TRAF6* mRNA to reduce TRAF6 protein expression	Cervical cancer, hepatocellular carcinoma, colorectal cancer	[195,197,198]
*miR-146a-5p*	Inhibition of TRAF6 expression	Pancreatic cancer, Non-small cell lung cancer	[199,208]
*miR-146b-5p*	Inhibition of TRAF6 expression	Colorectal cancer, glioblastoma	[200,201]
*miR-361-3p*	Inhibition of TRAF6 expression	Multiple myeloma	[202]
*miR-429*	Targeting the 3′UTR region of *TRAF6* mRNA to reduce TRAF6 protein expression	Hepatocellular carcinoma	[203]
*miR-605-3p*	Inhibition of TRAF6 expression	Hepatocellular carcinoma	[204]

## 7. Conclusions

TRAF6 is a pivotal adaptor protein with E3 ubiquitin ligase activity that predominantly acts as an oncoprotein in cancer biology, yet exerts tumor suppressive functions in a limited subset of malignancies [125]. TRAF6 activates TAK1 via K63-linked polyubiquitination, thereby constitutively driving the NF κB and MAPK signaling pathways to promote inflammation, tumor cell survival, and chemoresistance [31]. Concurrently, it participates in the activation of the PI3K/AKT pathway to support tumor angiogenesis and cell proliferation [133,134]. Critically, TRAF6 restricts the mitochondrial translocation and pro-apoptotic function of p53 by mediating its K63-linked ubiquitination, and blocks the interaction of RIPK1 with FADD/caspase 8, thereby inhibiting death receptor-mediated apoptosis and necroptosis [50]. Under normal physiological conditions, however, TRAF6 also performs protective functions such as maintaining genomic stability and regulating T cell receptor signaling. This dual functionality presents both challenges and opportunities for the development of selective targeted therapies.

Based on the mechanisms, targeted inhibition of TRAF6 pro-tumorigenic signaling complexes represents the primary therapeutic strategy. TRAF6 exerts its pro-tumor effects primarily through interactions with upstream receptors and downstream signaling molecules. Accordingly, the development of small-molecule inhibitors or peptide mimetics that can specifically disrupt these pro-tumorigenic protein–protein interactions may block NF-κB and MAPK pathways. Meanwhile, considering the complex role of TRAF6 in immune regulation, therapeutic strategies should focus on inhibiting its immunosuppressive functions. To mitigate the potential toxicity of systemic inhibition and improve the therapeutic effects, the development of tumor-specific delivery systems becomes particularly important. Future efforts should focus on designing antibody–drug conjugates or nanoparticles targeting tumor-associated antigens to enable the precise delivery of TRAF6 inhibitors to tumor tissues. Moreover, strategies aimed at selectively activating TRAF6′s anti-tumorigenic pathways may complement targeted inhibition of its pro-tumor activities. This includes the development of agonists that promote TRAF6-mediated activation of apoptotic signaling. Finally, given the complexity of TRAF6’s dual roles and the complexity of tumor signaling networks, combination therapies that target multiple nodes of TRAF6-dependent signaling may be more effective than single-agent approaches. Combining the TRAF6 inhibitor with a chemotherapeutic agent can synergistically induce cancer cell death.

Collectively, the development of TRAF6-targeted therapeutics should focus on four directions: (1) targeted inhibition of TRAF6 pro-tumorigenic signaling complexes to suppress oncogenic NF-κB and MAPK activation; (2) selective activation of TRAF6 anti-tumorigenic pathways to harness its pro-apoptotic and immune-regulatory potential; (3) tumor-specific delivery systems to enhance therapeutic precision; and (4) rational combination therapies to synergistically modulate multiple nodes within TRAF6-dependent signaling networks. By integrating precise drug delivery strategies and strategically designing combination regimens, the prospects of targeted TRAF6 intervention for overcoming treatment resistance and enhancing anti-tumor efficacy are substantially enhanced.

## Figures and Tables

**Table 1:** The substrates of TRAF6.

Substrates	Modifying	Regulatory Mechanism	Refs.
TAK1	K63	Stabilizing TAK1 to promote the NF-κB and MAPK signaling	[31]
Beclin 1	K63	Stabilizing Beclin 1 to promote autophagy	[32,33]
AKT	K63	Stabilizing AKT to activate the AKT pathway	[34]
MST1	K63	Activating MST1 to inhibit the NF-kB signaling	[35]
	/	Degrading MST1 to promote the Hippo signaling	[36]
YAP1	K63	Stabilizing YAP1 to promote the YAP1-TFCP2 signaling	[9]
IRF3	K48	Degrading IRF3 to promote the NF-κB signaling	[7]
IRF5	K63	Activating MyD88-dependent TLR signaling	[37]
IRF7	K63	Stabilizing IRF7 to induce the transcription of IFN-α	[38,39]
STAT3	K63	Promoting STAT3 phosphorylation and inhibiting its activity	[40,41,42]
STAT6	K63	Stabilizing STAT6 to promote the TLR signaling	[43]
MYC	K63	Inhibiting the transcriptional activity of MYC	[44]
HDAC3	K63	Inhibit the interaction between HDAC3 and c-Myc	[45]
β-catenin	K63	Promoting the autophagic degradation of β-catenin	[46]
ATG9A	K48/K63	Regulating the membrane localization and degradation of ATG9A	[30]
TAGLN	K48	Degrading TAGLN to promote the NF-κB and Myc signaling	[47]
FLT3	K48	Degrading FLT3 to inhibit the mTOR/ULK1 signaling	[48]
PIK3CA	/	Stabilizing PIK3CA to activate the AKT pathway	[49]
RIPK1	K48	Degrading RIPK1 to inhibit necroptosis	[50]
GPX4	K63	Promoting the autophagic degradation of GPX4	[51]
NFAT5	K63	Promoting the autophagic degradation of NFAT5	[52]
TXNIP	/	Stabilizing TXNIP to inhibit the growth of lung cancer	[53]
EZH2	K63	Degrading EZH2 to inhibit the malignant progression of tumors	[54,55,56]
BSG	K63	Stabilizing BSG and regulating the membrane transfer of BSG	[57,58]
hDNA2	K27/K63	Stabilizing hDNA2 to promote DNA replication	[59]
HK2	K63	Promoting the autophagic degradation of HK2	[60]
IRAK-1	K63	Ubiquitinating IRAK-1 to activate NF-κB signaling	[61]
LAT	K63	Ubiquitinating LAT to activate the TCR-LAT signaling	[62]
NRIF	K63	Ubiquitinating NRIF to promote its nuclear translocation	[63]
AEP	K63	Ubiquitinating AEP to promote the binding of AEP to HSP90α	[64]
MLK3	K63	Activating MLK3 to promote β-cell survival	[65]
mTOR	K63	Activating mTORC1 to inhibit autophagy	[66]
TβRI	K63	Activating TβRI to promote the TGFβ signaling	[67]
DJ-1	K6/K27/K29	Promoting the accumulation of mutant DJ-1 in the cytoplasm	[68]
PS1	K63	Stabilizing PS1 to endoplasmic reticulum calcium signaling	[69,70]
PINK1	K63	Stabilizing PINK1 to promote mitophagy	[71]
Rip2	K63	Stabilizing Rip2 to promote NOD2-mediated NF-κB activation	[72]
ASK1	K6	Activating ASK1, thereby intensifying the inflammatory	[73]
GSK3β	K63	Enhance the activity of GSK3β and promote its nuclear entry	[74]
	/	Degrading GSK3β to activate the Wnt signaling	[75]
CTLA-4	K48	Degrading CTLA-4 and enhancing the antitumor immune function	[76]
PRMT5	K63	Enhancing the methyltransferase activity of PRMT5.	[77]
ULK1	K63	Stabilizing ULK1 to promote autophagy	[78]

## Data Availability

No new data were created or analyzed in this study.
